# Supplementary material for: Enhancement of AFB1 Removal Efficiency via Adsorption/Photocatalysis Synergy Using Surface-Modified Electrospun PCL-g-C3N4/CQDs Membranes
Source: Biomolecules. 2023 Mar 17;13(3):550. doi: 10.3390/biom13030550 (PMC10046413; doi:10.3390/biom13030550)
Supplement: Supplementary file 1 [file biomolecules-13-00550-s001.zip › biomolecules-2191605-supplementary.pdf]

biomolecules

MDPI

Supplementary Information

Enhancement of AFB_1 Removal Efficiency via Adsorption/ Photocatalysis Synergy Using Surface-Modified Electrospun PCL-g-C_3N_4/CQDs Membranes

Liangtao Yao [1], Changpo Sun [2], Hui Lin [1], Guisheng Li [3], Zichao Lian [3], Ruixin Song [1], Songlin Zhuang [1] and Dawei Zhang [1,4,*]

[1] Engineering Research Center of Optical Instrument and System, Ministry of Education and Shanghai Key Lab of Modern Optical System, University of Shanghai for Science and Technology, No.516 Jungong Road, Shanghai 200093, China
[2] Standards and Quality Center of National Food and Strategic Reserves Administration, No.25 Yuetan North Street, Xicheng District, Beijing 100834, China
[3] Department of Chemistry, College of Science, University of Shanghai for Science and Technology, No.516 Jungong Road, Shanghai 200093, China
[4] Fujian Provincial Key Laboratory for Advanced Micro-Nano Photonics Technology and Devices, Research Center for Photonics Technology, Quanzhou Normal University, Quanzhou 362000, China
* Correspondence: dwzhang@usst.edu.cn

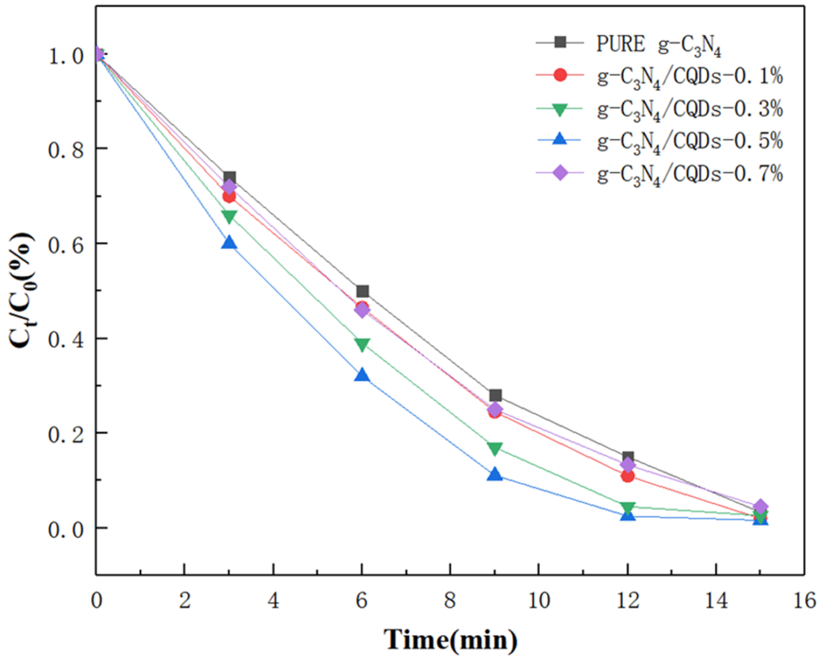

Figure S1. Photocatalytic degradation of Rhodamine B with different weight ratios of g-C_3N_4 and CQDS.

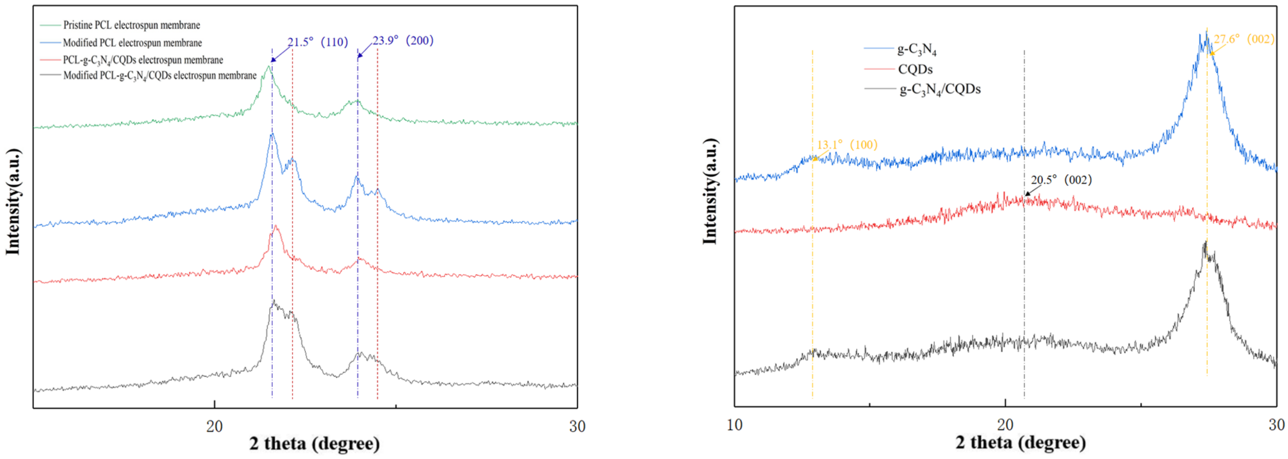

(**a**) (**b**)

Figure S2. XRD patterns of (a) four membranes from 15° to 30° and (b) g-C_3N_4, CQDs, g-C_3N_4/CQDs from 10° to 30°.

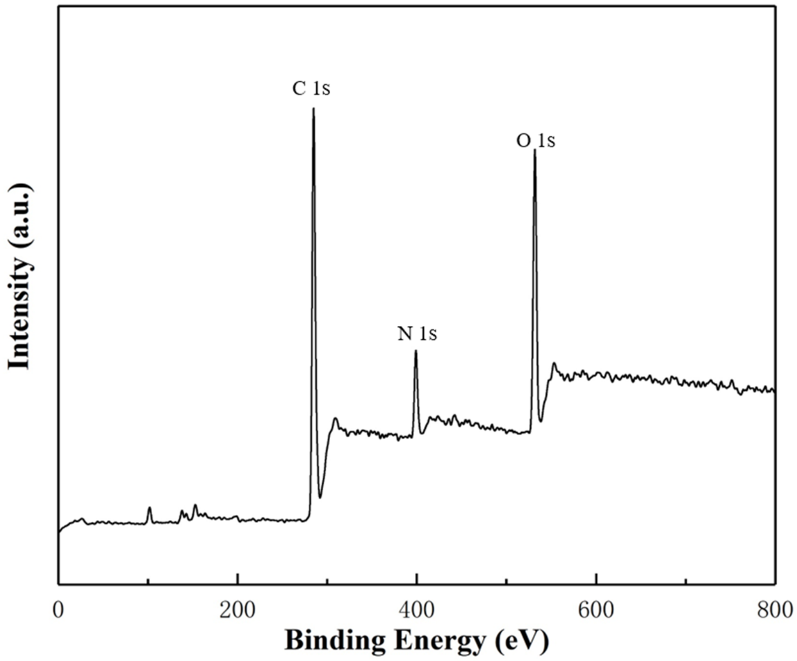

Figure S3. XPS survey spectrum of the modified PCL-g-C_3N_4/CQDs electrospun membrane.

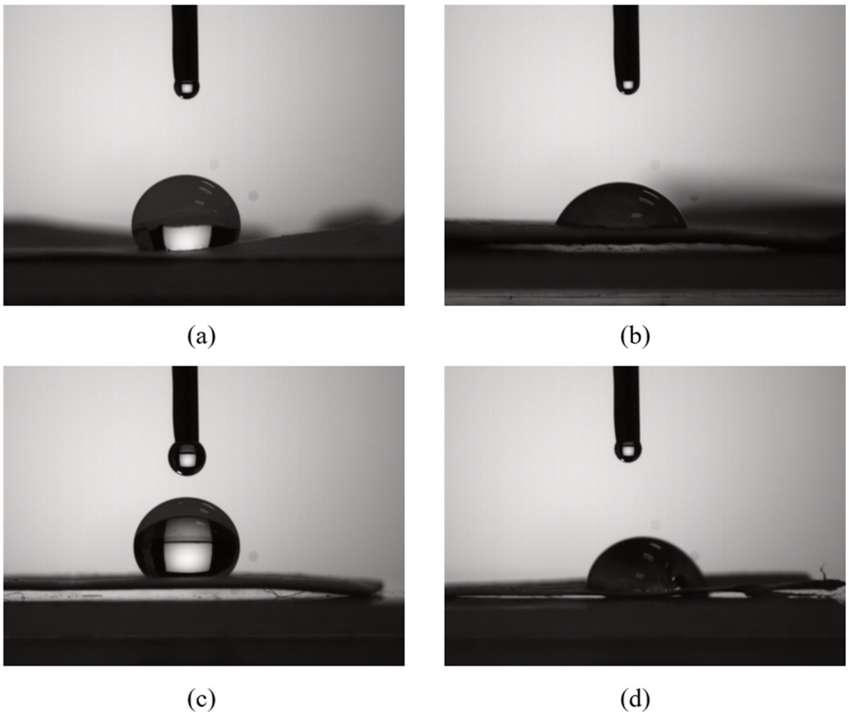

Figure S4. Optical images taken while the water droplets come into contact to the surface of (**a**) pristine PCL electrospun membranes (125.59°), (**b**) modified PCL electrospun membranes (64.03°), (**c**) PCL-g-C_3N_4/CQDs electrospun membranes (129.49°), and (**d**) modified PCL-g-C_3N_4/CQDs electrospun membranes (76.55°).
